# Beyond Refeeding: The Effect of Including a Dietitian in Eating Disorder Treatment. A Systematic Review

**DOI:** 10.3390/nu13124490

**Published:** 2021-12-15

**Authors:** Yive Yang, Janet Conti, Caitlin M. McMaster, Phillipa Hay

**Affiliations:** 1 Translational Health Research Institute, School of Medicine, Western Sydney University, Campbelltown, NSW 2560, Australia; yive.yang@westernsydney.edu.au (Y.Y.); j.conti@westernsydney.edu.au (J.C.); 2 School of Psychology, Western Sydney University, Penrith, NSW 2750, Australia; 3 University of Sydney Children’s Hospital at Westmead Clinical School, Faculty of Medicine and Health, Westmead, NSW 2145, Australia; caitlin.mcmaster@sydney.edu.au; 4 Camden and Campbelltown Hospitals, South Western Sydney Local Health District, Sydney, NSW 2560, Australia

**Keywords:** feeding and eating disorders, dietetics, nutrition counselling, nutrition therapy, outpatient

## Abstract

Eating disorders are potentially life-threatening mental health disorders that require management by a multidisciplinary team including medical, psychological and dietetic specialties. This review systematically evaluated the available literature to determine the effect of including a dietitian in outpatient eating disorder (ED) treatment, and to contribute to the understanding of a dietitian’s role in ED treatment. Six databases and Google Scholar were searched for articles that compared treatment outcomes for individuals receiving specialist dietetic treatment with outcomes for those receiving any comparative treatment. Studies needed to be controlled trials where outcomes were measured by a validated instrument (PROSPERO CRD42021224126). The searches returned 16,327 articles, of which 11 articles reporting on 10 studies were included. Two studies found that dietetic intervention significantly improved ED psychopathology, and three found that it did not. Three studies reported that dietetic input improved other psychopathological markers, and three reported that it did not. One consistent finding was that dietetic input improved body mass index/weight and nutritional intake, although only two and three studies reported on each outcome, respectively. A variety of instruments were used to measure each outcome type, making direct comparisons between studies difficult. Furthermore, there was no consistent definition of the dietetic components included, with many containing psychological components. Most studies included were also published over 20 years ago and are now out of date. Further research is needed to develop consistent dietetic guidelines and outcome measures; this would help to clearly define the role of each member of the multidisciplinary team, and particularly the role of dietitians, in ED treatment.

## 1. Introduction

Eating disorders (EDs) are multidimensional and potentially life-threatening disorders that involve complex psychosocial issues. Symptoms of EDs include problematic eating, exercise behaviours and body image that contribute to impairment in mood and quality of life [1,2,3,4]. The lifetime prevalence of EDs in the general population is approximately 8.4% for women and 2.2% for men; in 2019, the disability-adjusted life years (i.e., years of healthy life lost to mortality or disability) totalled 6.6 million for EDs [5,6]. Furthermore, a 4.3% rise in ED point prevalence from the 2000–2006 period to the 2013–2018 period highlights the increasing challenge that EDs present for public health and healthcare providers [6].

In order to optimise the chances of a full recovery from an ED, a multidisciplinary approach including medical, psychological and dietetic involvement is recommended by multiple practice guidelines across Australia, the United Kingdom, and the United States [1,7,8,9]. Enhanced cognitive behavioural therapy (CBT-E), the most extensively researched and validated modality for ED treatment, also suggests that consultation with a dietitian may be beneficial [10,11]. To date, dietitians are consistently included as part of the treatment team for EDs.

Whilst dietitians are viewed as critical members of multidisciplinary ED treatments, there is a need for a more detailed and wider understanding about the contribution and efficacy of dietetic intervention in these treatments. A dietitian’s expertise centres around their specialised knowledge and skills regarding nutritional science and behaviour change to aid in nutritional rehabilitation (i.e., medical stabilisation via refeeding, weight goals, and achieving adequate and appropriate nutritional intake). Nevertheless, there is a paucity of research on the unique contributions of a dietitian in ED treatment outcomes as well as treatment experiences [12,13,14,15,16,17]. Furthermore, current dietetic guidelines are primarily based on the ‘expert opinion’ of clinicians, with some guidelines recommending nutritional intervention without consulting a dietitian [15]. The most recent practice and training standards for dietitians working with EDs published by the Australian and New Zealand Academy for Eating Disorders (ANZAED) aimed to identify and describe the role of dietitians in ED treatment [18]. Whilst providing perhaps the most comprehensive role statement to date and providing an outline of the skills and knowledge a dietitian working in ED treatment possesses, it must be noted that these recommendations were based only on the consensus opinions of expert dietitians and other health professionals in the ED field.

There is also little research addressing the overlap between the role of the dietitian and the role of the psychologist in ED treatments, and the effect that this may have on treatment outcomes [19]. Joy et al. (2003) observed that whilst each member of the multidisciplinary team (MDT) has unique skills and knowledge regarding ED treatment, there is substantial overlap in what each member provides to support recovery from an ED [20]. Therapies such as CBT-E, whilst suggesting consultation with a dietitian, do not provide a clear role for nutrition counselling by a dietitian, and also contain elements of nutritional counselling that may be administered by psychotherapists alone [10]. Family based treatment (FBT) for children and adolescents with EDs also does not specify a role for the dietitian, although suggests that if the primary therapist does not have adequate nutrition knowledge, a dietitian “could be beneficial” [21]. There has been research into FBT that suggests that some therapists are uncomfortable performing FBT without a dietitian [22]. In outpatient practice, dietitians are also playing an increasingly active and important role in FBT treatment and there are resources and training opportunities targeted at dietitians to upskill in this area [23,24,25].

Thus, there is ongoing ambiguity about the specific roles of (1) the dietitian in EDs and how this complements/overlaps with psychotherapy; (2) the psychologist/psychotherapist and how this complements/overlaps with dietetic treatment. One explanation could be the lack of evidence of the effectiveness of dietetic input in ED treatments [17]. A recent systematic review by McMaster et al. examined the available evidence for dietetic intervention in adult outpatients with an ED [17]. In their review, McMaster et al. highlighted the lack of available evidence to determine the impact of dietetic intervention on ED treatment [17]. Whilst this review is current, it did not include studies that examined EDs outside of anorexia nervosa (AN), bulimia nervosa (BN), binge eating disorder (BED) and other specified feeding or eating disorders (OSFED) and excluded studies on children and adolescents. Perhaps most importantly, McMaster et al. included studies where the nutrition intervention was not conducted by a dietitian and did not focus on the professional role of a dietitian beyond nutritional interventions. Therefore, the evidence is incomplete, and there is a need to specifically determine what the effects and specific roles of a dietitian are in order to optimise the utility of dietitians in ED management.

This systematic review aims to determine the effect of the inclusion of a clinician from the discipline of dietetics (e.g., in Australia, an Accredited Practicing Dietitian (APD); in the U.S., a Registered Dietitian) where the clinician is providing specialist dietetic care, on outpatient ED treatment outcomes, and to contribute to the understanding of the role of dietitians in ED treatment. It should be noted that accreditation and registration for dietitians varies internationally. In Australia, there is registration with Dietitians Australia, which accredits APDs. Whilst there are other health professionals, such as nutritionists, who may provide nutritional advice, those who have not met the criteria to be an APD are unlikely to be working with EDs and cannot access government rebates for outpatient ED treatment. This review focuses on dietetic care as would be provided by an APD in Australia. This review also focuses on the role of a dietitian in outpatient treatment, as the role of a dietitian in inpatient treatment to help achieve nutritional stabilisation and medical refeeding is well documented [7,9].

## 2. Materials and Methods

### 2.1. Search Strategy and Selection Criteria

A search strategy was constructed with advice from the Western Sydney University medical librarian. Key terms from three categories relevant to this review were identified. These were (1) EDs, (2) dietitians and (3) treatment (see Appendix A Table A1 for full search terms). Search terms from each of the three categories were combined as follows: ((words related to ED) AND (words related to dietitians) AND (words related to treatment)); truncation was used for key terms. The searches were conducted on the 28 April 2021. The protocol was registered with the international prospective register of systematic reviews (PROSPERO), in accordance with PRISMA-P guidelines (PROSPERO CRD42021224126).

The search involved identifying published journal articles, guidelines and grey literature that considered the role of a dietitian in ED treatment. Electronic databases searched were MedLine, EMBASE, Scopus, ProQuest Dissertation and Thesis, the Cumulative Index to Nursing and Allied Health Literature (CINAHL) and Cochrane Collaboration Database. The searches were from first date of the respective database to the present. Google Scholar was searched for grey literature. The phrases “dietitian eating disorder treatment” and “dietitian eating disorder treatment role” were entered into Google Scholar, and articles from the first 10 pages of each search (i.e., first 100 articles from each search) were included. The reference lists of included articles were also searched. The full search strategy is presented in Table S1 in the Supplementary files.

Citations and abstracts were exported to COVIDENCE [26], and duplicates excluded. The title and abstract of each paper were screened by one reviewer (Y.Y.) for their relevance and adherence to a broad inclusion criterion. Papers were included if they focused exclusively or partially on the role of dietitians regarding treatment for any type of ED. Papers were excluded if they were published in any language other than English, did not include dietitians as part of the treatment team for an ED, did not distinguish the data regarding the dietitian from that regarding the rest of the MDT, only focused on medical complications or medical stabilisation, were not original research (i.e., other systematic reviews), were an abstract only, were an incomplete study protocol, or were an earlier version of an included paper. Here, any duplicates that were missed by COVIDENCE were manually removed. Two reviewers (P.H., J.C.) each checked ten percent of screened titles for consistency.

One reviewer (Y.Y.) then read the final full-text articles, and a second selection was made to keep only articles that met a second inclusion criteria. Articles were included if they were a controlled study, included specialist dietetic care as part of the ED treatment team, included outcomes measured by a validated instrument or questionnaire, and were conducted in an outpatient setting (see Appendix B Table A2 for PICO criteria) [27]. Two reviewers (P.H. and J.C.) each checked 25% of screened full texts for consistency. Discrepancies at each stage were noted and resolved by a third reviewer (P.H. or J.C.).

### 2.2. Data Extraction and Analysis

Two data extraction tables were created using Microsoft Excel (Microsoft Cooperation, 2019, Seattle, WA, United States) and Microsoft Word (Microsoft Cooperation, 2019, Seattle, WA, United States). Data collected were author, country of study, study design (see below for the quality appraisal), participant characteristics, description of dietetic intervention, and main treatment outcomes. One reviewer (Y.Y.) assessed the quality of all papers and collected all data. Two other reviewers (P.H. and C.M.M.) independently conducted data collection and quality appraisal of 30% of the included papers each. Any discrepancies in ratings or data collected were resolved through consensus discussion. Data were synthesised narratively as the studies included used different intervention and outcome measures, which did not allow for data pooling for meta-analyses.

### 2.3. Quality Appraisal and Risk of Bias

The quality of articles included was assessed using the revised Mixed Methods Appraisal Tool (MMAT 2018) [28]. Item 2.4 in the MMAT (“Are assessors blinded to the intervention provided?”) was amended to include blinding of participants and researchers where appropriate as this could also contribute to study bias.

## 3. Results

### 3.1. Study Selection

**Figure 1:**
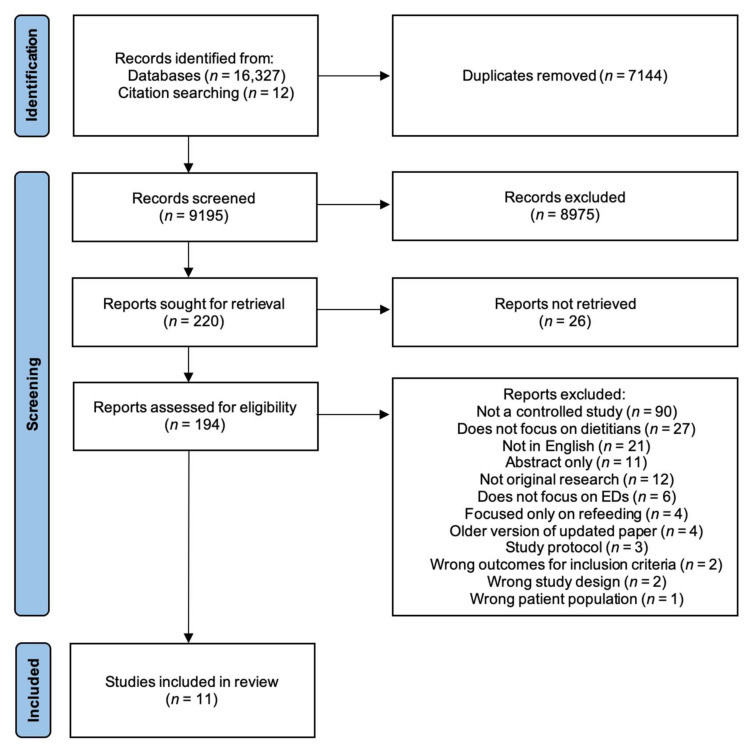
Identification and selection of articles included.

A total of 16,339 studies were identified, 16,327 from databases and Google Scholar, and 12 from searching the reference lists of included studies. Of these papers, 11 fulfilled the inclusion criteria and were included in the present review [29,30,31,32,33,34,35,36,37,38,39]. Eight of these were randomised controlled trials [29,30,33,34,35,36,38,39], and three were non-randomised controlled trials [31,32,37]. A further two of these papers reported on the same trial [31,32], and thus were combined and counted as one study for analysis. Figure 1 details the PRISMA 2020 flow diagram for the search and study selection process [40].

### 3.2. Study Characteristics

The descriptive characteristics of included studies [29,30,31,32,33,34,35,36,37,38,39] are outlined in Table 1.

#### 3.2.1. Participant Characteristics

Sample sizes ranged from 30–189 participants, with a total sample size of 682. Of these, 581 (85%) were female, and three studies included males [31,32,36,37]. The mean age of participants ranged from 19.56 to 51.1 years old, although only two studies included participants whose mean age was greater than 25 years old, and both these studies reported on a BED-only population [30,31,32]. Three studies were performed in people with AN only [33,36,37], three performed in participants with BN only [34,35], and one study included participants with both AN and BN [29]. Total participant attrition rate ranged from none to 34%.

#### 3.2.2. Dietetic Intervention Characteristics

All studies included at least one intervention arm involving dietetic treatment performed by a dietitian, except one [31,32], which was included on the assumption that the intervention was delivered by a dietitian based on intervention components. The components of the dietetic arm of each study are outlined in Table 2. Participants from seven studies [29,30,33,34,36,37,39] received individual dietetic treatment, two studies provided group treatment [35,38], and one study provided combined individual and group treatment [31,32]. Duration of intervention ranged from 3 months to 12+ months. Attrition rate for the dietetic arm ranged from none to 100%. In the study by Serfaty et al. [37], all dietetic intervention group participants dropped out and analyses of outcomes could not be conducted. Therefore, this study was removed from the study outcomes reported below. The dietetic interventions in six of the included studies [31,32,33,35,36,37,39] also contained therapeutic treatment outside of traditional nutritional care.

#### 3.2.3. Comparator Intervention Characteristics

The comparator intervention in most studies was some form of psychological treatment performed either concurrently with, or separate to, the dietetic intervention. One trial included medication as part of the psychological intervention [30], one study included dietetic intervention in the psychological arms [29], one study included physical exercise as an additional intervention [38], one study compared two types of dietetic intervention [39], and one study compared dietetic intervention with combined dietetic intervention and fluoxetine [36]. Participants from six studies [29,33,34,36,37,39] received individual treatment, two studies provided group treatment [35,38], and two studies provided a mix of individual and group treatment [30,31,32], as the comparator. Comparator intervention duration ranged from 3 months to 12+ months. Attrition rate for the comparator arm of the studies ranged from 0 to 29%.

### 3.3. Study Outcomes

The results are summarised in Table 3.

#### 3.3.1. ED Psychopathology

Most studies reported on ED psychopathology [29,30,31,32,33,34,35,36,37,38], and those that reported the measure used a validated instrument. Overall, two studies found that dietetic intervention significantly improved ED psychopathology [35,36], three studies found that dietetic intervention did not significantly improve ED psychopathology [29,30,31,32], and four studies did not report within-group results [33,34,38,39]. In three studies, psychological therapy was found to perform superior to dietary counselling in reducing some ED psychopathology [33,34,38]. In two studies, significant reductions in ED psychopathology were seen in the psychological intervention group but not in the dietary counselling group (between groups not reported) [30,31,32]. Nutritional advice was found to be superior to psychotherapy in improving bulimia, vomiting, and purgation symptoms by Hall et al. [33]. Ruggiero et al. found that nutritional treatment achieved significant decreases in fear of fatness in the study, whilst combined nutritional treatment and fluoxetine did not [36].

Hsu et al. reported that combined cognitive nutritional therapy also performed better than nutritional therapy alone in reducing “drive for thinness”, “bulimia” and “ineffectiveness”, but there was no difference between combined cognitive nutritional therapy and cognitive therapy alone. A similar reduction in ED symptoms was reported for both stress management and a dietetic intervention, by Laessle et al. [35].

#### 3.3.2. Other Psychopathology

Seven studies reported on other psychopathology outcomes using a validated instrument [29,30,31,32,33,34,35,37]. Of the three studies that did not report other psychopathology, two [36,39] had dietetic interventions as part of both study arms. Overall, three studies [33,34,35] found that dietetic intervention significantly improved other psychopathology and three studies [29,30,31,32] found that dietetic intervention did not significantly improve other psychopathology. Between-group differences were not reported in three studies [30,31,32,35]. Bachar et al. and Hall et al. reported no significant difference between dietetic intervention and comparators (either psychological intervention or psychological intervention combined with dietetic intervention), and neither reported on within-group changes. Combined cognitive nutritional therapy was observed by Hsu et al. [34] to improve dysfunctional attitudes more than nutrition therapy alone did, and there was also no difference between combined cognitive nutrition therapy and cognitive therapy alone.

#### 3.3.3. Level of Function and Quality of Life Measures

Only four studies reported on level of function/quality of life [31,32,33,34,37]. Hall et al. [33] reported that psychotherapy was better than dietary advice at improving social and sexual adjustment scores. Whilst dietetic intervention, cognitive therapy, combined cognitive nutritional therapy, and support groups all improved self-control scale scores, Hsu et al. [34] found that dietetic and comparator groups both improved more than the support group, and combined cognitive nutritional therapy performed better than dietetic intervention alone.

#### 3.3.4. ED Behaviours/Weight

Nine studies measured changes in ED behaviour/weight [30,31,32,33,34,35,36,37,38,39]. Four studies reported on either weight or body mass index (BMI) changes, of which three observed participants with AN [33,36,37], and one observed participants with BED [31,32]. In the BED group, all interventions resulted in weight decrease [31]. Hall et al. [33] observed an increase in weight in the dietary advice group only but did not report between-group differences. Ruggiero et al. observed an increase in BMI in both nutritional management and combined nutritional management with fluoxetine groups, and the group with added fluoxetine had a greater increase. Six studies reported on binge frequency [30,31,32,34,35,38,39], and four studies reported on vomiting frequency [34,35,38,39]. Of those that reported binge frequency, three studies [34,35,39] observed significant reductions in the dietetic arm, one study [30] observed no significant change in the dietetic arm, and two studies did not report within-group changes [31,32,38]. Ventura et al. [39] found that psychobiological nutrition rehabilitation reduced binge and vomiting frequency more than traditional nutrition rehabilitation did, whilst Hsu et al. [34] and Laessle et al. [35] reported no between-group differences, although in both studies, psychological and nutritional interventions significantly reduced binge and vomiting frequency. Three studies further reported on vomiting frequency [34,35,38,39]. Sungot-Borgen reported that cognitive behavioural therapy (CBT) reduced vomiting frequency more than nutritional counselling did.

#### 3.3.5. Diet Quality/Diet Adequacy

Only three studies reported on diet adequacy/quality [34,35,39], and none used a validated instrument. Only Ventura et al. [39] found between-group differences in diet quality/adequacy, and they reported that psychobiological nutritional therapy was better than traditional nutritional rehabilitation at increasing lipid intake (as measured by the number of olive oil servings added to the diet) in individuals with BN. Hsu et al. [34] and Laessle et al. [35] both observed significant increases in food intake (measured by meal number or caloric intake outside of binge episodes) within groups for both psychological and dietetic interventions.

### 3.4. Quality Appraisal and Risk of Bias

The results of the quality appraisal are summarised in Table 4. Most included RCTs (*n* = 7) did not report if randomisation was appropriately performed [29,30,33,34,35,38,39], and blinding was either not appropriately conducted or unclear in seven RCTs [29,33,34,35,37,38]. Only one of two non-randomised studies adjusted appropriately for confounders [31,32]. Adherence to assigned intervention was also either not reported or not clearly reported in most papers (*n* = 7) [29,30,31,32,33,35,36,37,38]. A majority of included studies had complete outcome data (*n* = 8) [29,30,33,34,35,38,39], defined as attrition of less than 29% [41]. Whilst most papers collected data that adequately addressed the research questions (*n* = 8) [29,30,31,32,33,34,35,36,39], outcome measures were highly varied and non-standardised across studies.

## 4. Discussion

### 4.1. Summary of Findings

This review systematically evaluated the effect of including a dietitian in outpatient ED treatment in studies that included a validated instrument. There were no restrictions placed on participant age, type of ED, or publication date. Despite the broad inclusion criteria, only ten studies (11 publications) were identified, thus reflecting the paucity of research in this area, mirroring previous reviews [13,17]. Whilst all studies used at least one validated instrument to measure outcomes, these were not standardised across studies and a large variety of tools were used, thus making direct comparison between studies difficult.

The results of the studies were mixed. In regards to ED psychopathology, two studies [35,36] found that dietetic intervention significantly improved symptoms, whilst three studies did not [29,30,31,32]. Similarly, for other psychopathology, three studies [33,34,35] indicated that dietetic intervention significantly helped, whilst three others found that it did not [29,30,31,32]. An explanation for these inconsistencies could be the wide variation in the dietetic intervention components provided. Whilst some studies detailed the topics that were discussed and what was entailed in dietetic treatment [29,31,32,34,35,36,39], other studies had broad definitions of dietetic intervention or only brief descriptions [30,33,37,38], such as Brambilla et al., who prescribed “nutritional advices but not a specific diet” in their dietetic arm. This issue was similarly noted in the systematic review by McMaster et al. [17]. Additionally, McMaster et al., in their review of psychological treatment manuals for adults with EDs, observed a lack of a cohesive understanding of what dietetic intervention entails and reported that whilst 91% of manuals contained nutrition and food-related content, 60% of manuals contained content not substantiated by current research evidence [15]. Without a research evidence-based, unified, and consistent description of what dietetic intervention is, it is very difficult to determine the effect of dietetic intervention in EDs and provide recommendations for dietitians working in this field. 

One consistent finding was that BMI/weight in participants with AN was seen to increase significantly with dietetic input [33,36], although it is noted that this was only reported by two studies. Similarly, all studies that examined some aspect of diet quality or adequacy found that dietetic intervention significantly approved nutritional intake [34,35,39]. These findings are likely due to the dietitian’s expertise and well-established role in helping individuals improve the nutritional adequacy of their diet [12,13,14,18]. Despite this, nutritional outcomes measuring diet quality and adequacy were reported by only three papers [35,39,42], and these did not use a validated instrument. In the treatment of EDs, it is crucial to consider dietary quality and adequacy as individuals who experience EDs will continue to be nutrient-deficient, have altered nutrient intake profiles, and exhibit restrictive eating behaviours with decreased diet variety and persistent food rules, even after weight restoration [43,44,45,46].

### 4.2. Intervention Components

Only three studies [29,31,32,34] included combined psychological and nutritional intervention as one arm of the study, and of these, only two compared combined treatment against psychological treatment alone [31,32,34]. In these studies, combined nutritional and cognitive therapy significantly improved most outcomes. However, Hsu et al. found no significant difference between cognitive therapy and combined nutritional cognitive therapy in changing any outcomes [34]. The other studies included compared dietary intervention against another dietetic intervention, psychological intervention, combined dietetic and pharmacological intervention, combined pharmacological and psychological intervention, or physical exercise. By making these comparisons, most of the included studies assumed that dietetic intervention seeks to act as a replacement for psychological interventions. However, current guidelines do not advocate for dietetic treatment in the absence of psychological treatment, and best practice involves a MDT consisting of dietitians, psychologists, and psychiatrists [1,7,8,9]. Current research lacks an understanding of how dietetics improves ED outcomes and the treatment experiences of individuals with an ED, and the unique contribution dietitians make to treatment alongside psychological and medical interventions.

In six studies, dietitians in the dietetic intervention arm provided intervention components outside of nutritional care, or components acknowledged by recent practice standards as core aspects of treatment which should be delivered by any ED clinician, regardless of their discipline [47]. Aspects of care included psychoeducation about the relationship between restrained eating and bingeing [35], psychological support using supportive counselling [37], and long-term psychoeducational treatment that aimed to achieve weight restoration [36]. In particular, the study by Laessle et al. [35] proposed and researched a dietetic intervention, developed in Australia, that incidentally consisted of several therapeutic components also contained in the first wave of CBT for BN, concurrently developed by Fairburn et.al. (1993) in the UK [48]. The comparison group of stress management in this study also contained other non-nutritional components that are included in CBT-E, and the treatment outcomes for participants in this group were similar to those for participants in the dietetic intervention group [10]. At present, the delineation between the components of ED treatment delivered by a dietitian and those components delivered by a psychologist is unclear [20]. This ambiguity further contributes to the lack of clarity around defining dietetic intervention components and precipitates the need for a consistent understanding of these components in order to evaluate the efficacy and effectiveness of dietitians in ED treatment.

### 4.3. Strengths and Limitations of Included Studies

Across all but one of the randomised trials included in this paper, randomisation was not adequately described, and only one study blinded participants, researchers and outcome assessors to the intervention provided. Thus, inappropriate randomisation and blinding contributed significantly to overall risk of bias [42,49]. In the study conducted by Serfaty et al. [37], all participants from the dietetic study arm dropped out. Whilst authors suggested that this may have been due to differences in severity of illness at baseline, another plausible explanation could be the lack of participant blinding leading to performance bias, and participants dropping out due to preference for the primary intervention. Previous literature has suggested that dietetic input for an ED is perceived by healthcare practitioners and individuals with lived experience to be both important and helpful, and sometimes even more so than input from a psychologist or psychiatrist [50,51,52]. However, evidence has shown that participant knowledge of group assignment can increase attrition and use of co-intervention, especially in the control group, which then skews results [42,53]. The findings of Serfaty et al. [37] underscore the importance of replication, as no other study has resulted in 100% attrition in the dietetic arm, which could be interpreted as a negative result. In addition, two included studies [31,32,36] were non-randomised, and only one of these [31,32] accounted appropriately for confounders, thus increasing risk of bias in the study that did not. Furthermore, only three studies [34,35,37] performed an intention-to-treat analysis, without which can also lead to an increased risk of bias.

One study [31,32] did not explicitly specify if the nutritional intervention components were carried out by a dietitian, and the authors did not respond when contacted. However, based on the specific meal planning, psychotherapy on weight and nutrition, as well as the nutritional assessment provided, reviewers assumed that the nutritional intervention was carried out by the equivalent of an APD in Australia. There are healthcare professionals, such as nutritionists, that provide ‘nutritional care’ and who do not meet the training standards as set for those who are APDs, or Registered Dietitians in the U.S, and who do not routinely work with EDs. Psychologists are also able to provide some basic nutrition information in therapies such as CBT-E [10]. Future studies should clearly report the profession of the individual who performs nutrition therapy in order to remove ambiguity and allow for more accurate pooling of data.

The eleven papers on ten studies reported in this systematic review only reported on AN, BN, and BED, and only two studies examined BED [30,31,32]. Despite our broad inclusion criteria, no papers examining the role of a dietitian in other EDs classified under the DSM-5, such as OSFED, Pica, or rumination disorder, were captured in our search. BED and OSFED account for the majority of ED cases [5]. The lack of research on two of the most common EDs further highlights the need for more research to understand the effect of the dietitian in their treatment, and in other less common EDs. Furthermore, six of the ten studies included in this review were published over 20 years ago, and of the remaining, only one was conducted within the last 10 years [31,32]. In the study by Ventura et al. [39], two types of dietetic treatment were compared. These were traditional nutritional rehabilitation (TNR) and psychobiological nutritional rehabilitation (PNR). Aspects of PNR are working to identify hunger, appetite, and satiety cues, and focusing on how bodily functions control appetite and body weight. These ‘differentiating’ aspects of PNR are now commonly included in dietetic treatment and are even included in other studies included in this review [14,18,35,36]. This then undermines the need to compare what was described as ‘traditional’ dietetic treatment with PNR and highlights the need for new research to reflect changes in dietetic treatment.

### 4.4. Strengths and Limitations of This Review

The strengths of this present study include the registration of review protocol with PROSPERO, comprehensive search strategy, broad inclusion criteria, and inclusion of grey literature. Multiple authors also independently reviewed data extraction and performed quality appraisal. This review captured different literature to that in the systematic review by McMaster et al. [17], with three additional papers identified [30,36,39], as well as capturing different outcome measures. A key limitation was the small number of studies included (*n* = 10), and a meta-analysis could not be conducted due to the heterogeneity in study design and outcomes measures. Furthermore, whilst a proportion of records were screened by a second reviewer, there is the possibility that some relevant publications may have been missed by a single reviewer.

### 4.5. Implications for Future Practice and Research

This review builds on the findings of previous reviews and highlights the lack of homogenous study methodologies and intervention components in current literature around the effect of a dietitian in ED treatment [13,15,17]. Manualisation of dietetic treatment, similar to the manualisation of CBT-E and other psychotherapeutic models, would provide a benchmark of dietetic care for EDs, which could then be used to compare the effectiveness of ED intervention with and without a dietitian [10,54]. Use of standardised outcome measures would also allow for more meaningful comparisons to be made across studies. Furthermore, development of validated nutrition quality assessment tools, or adaption and validation of existing tools such as the Australian Eating Survey [55] or Dietary Questionnaire for Epidemiological Studies [56], specific to EDs could be routinely included as outcome assessment measures to determine if dietetic input improved food choices beyond only refeeding and reduction in other ED symptoms. Whilst previous studies have shown that dietary assessment tools in ED populations do not necessarily provide an accurate representation of energy intake, tool standardisation will allow for understanding of a dietitian’s impact on nutrition quality through comparison within, as well as across, studies [57,58].

Findings of this review support recent dietetic practice standards that demonstrate the role of dietitians in ED treatment beyond refeeding the underweight person, meal planning, and the provision of nutritional advice [18]. However, more research is required to quantify what difference dietetic input makes when added to psychological treatments. There is also a need for more clarity around what the scope of a psychologist or dietitian is in order to facilitate multidisciplinary collaboration in treatment as per clinical practice guidelines. Thus, it is suggested that future studies examine dietetic intervention alongside psychotherapy, and compare against psychotherapy alone, to understand if dietitians positively contribute to ED recovery. Better reporting of nutrition intervention components in future studies would also improve understanding of the effect of a dietitian. More research is required to not only add to the pool of available literature but also to update findings to reflect changes in dietetic practice.

## 5. Conclusions

Overall, the inconsistencies in findings and large variety in study methodologies and outcomes measures highlight the lack of quality, up-to-date research available regarding the effect of a dietitian in ED treatment. There is a need for studies that report consistent outcome measures and that use standardised and relevant treatment methods. Findings in this review suggest that the professional role of a dietitian in ED treatment extends beyond refeeding, meal planning, and provision of nutrition education alone. Dietitians are well placed to be able to intertwine their expertise around nutrition with psychotherapeutic modalities when working with other members of a MDT as part of ED treatment, and a dietitian may be able to provide primary care psychological therapy such as guided self-help. However, more research is required to define the role of a dietitian, beyond assisting with refeeding, to allow delivery of effective collaborative treatment.

## Figures and Tables

**Table 1:** Characteristics of included studies.

	**Participant Characteristics**			**Dietetic Intervention Group**			**Comparator Intervention Group(s)**			
**Study**	**Study** **Design, Country**	**Total Sample Size,** **Final Sample Size (% Drop Out), % Female**	**ED Diagnosis (*n*), Mean Age (SD)**	**No. (No. of Drop-Outs)**	**Mean Baseline BMI**	**Group/Individual, Duration**	**Intervention** **Type**	**No. (No. of Drop-Outs)**	**Mean** **Baseline BMI**	**Group/** **Individual, Duration**
Bachar 1999 [29]	RCT, Israel	44, 33 (25), 100	BN (25), 24.1 (SD = 3.3) AN (8), 18.1 (SD = 2.4)	10 (3)	NR	Individual, 6 months	SPT + NC COT + NC	17 (3) 17 (5)	NR	Individual, 12 months
Brambilla 2009 [30]	RCT, Italy	30, 30 (0), 100 Inconsistent reporting of sample size and drop-out.	BED (30), 42.9 (SD = 9)	10 (0)	34 (SD = 5)	Group CBT + assume diet component individual but NR, 6 months	Group 1: 1700 calorie macronutrient-controlled diet + CBT + sertraline (50–150 mg/d) + topiramate (25–150 mg/d) Group 2: 1700 calorie, macronutrient-controlled diet + CBT + sertraline (50–150 mg/d)	10 (0) 10 (0)	39 (SD = 6) 34 (SD = 6)	Group CBT, assume diet component individual but NR, 6 months Group CBT, assume diet component individual but NR, 6 months
Compare 2013 & 2016 [31,32]	Controlled observational study, Italy	189, 164 (13), 50	BED (189), EFT group: 50.8 (SD = 6.0); Combined therapy group: 51.1 (SD = 4.1); DT group: 50.4 (SD = 4.7)	63 (17)	32.3 (SD = 1.3)	Individual and group, 20 weeks	Emotion-focused therapy Combined therapy	63 (8) 63 (0)	33.0 (SD = 1.6) 33.6 (SD = 2.6)	Group, 5 months Group EFT, 5 months + Individual DT, 3 months
Hall 1987 [33]	RCT, UK	30, 25 (17), 100	AN (30), 19.56 (range 13–27)	15 (4)	Weight 39.54 kg	Individual, 12–24 weeks	Individual and family psychotherapy	15 (1)	Weight 41.0 kg	Individual, 12–24 weeks
Hsu 2001 [34]	RCT, USA	100, 73 (27), 100	BN (100), 24.2 (SD = 5.6)	23 (9)	NR	Individual, 14 weeks	Cognitive therapy CNT Support group	26 (4) 27 (3) 24 (11)	NR	Individual, 14 weeks
Laessle 1991 [35]	RCT, Australia and Germany	55, 48 (13), 100	BN (55), 23.8 (SD = 3.8)	27 (5)	21.2 (SD = 1.8)	Group, 3 months	Stress management	28 (2)	20.6 (SD = 1.9)	Group, 3 months
Ruggiero 2003 [36]	Non-randomised controlled trial, Italy	95, 95 (0), 96	AN (95), 23.47 (SD = 4.93)	74 (0)	14.29 (SD = 2.18)	Individual, 12 months+	Nutritional management + fluoxetine	21 (0)	14.83 (SD = 1.53)	Individual, 12 months+
Serfaty 1999 [37]	RCT, UK	35, 23 (34), 94	AN (35), 20.9 (SD = 6.3)	10 (10)	17.0 (SD = 4.0)	Individual, 20 weeks	Cognitive therapy	25 (2)	16.2 (SD = 1.6)	Individual, 20 weeks
Sundgot-Borgen 2002 [38]	RCT, Norway	64, 58 (9), 100	BN (64), 22.5 (SD = 2.8)	17 (0)	21.0 (SD = 2.1)	Group, 16 weeks	Cognitive behavioural therapy Physical exercise	16 (2) 15 (3)	20.0 (SD = 1.9) 21.0 (SD = 2.0)	Group, 16 weeks Group, 16 weeks
Ventura 1999 [39]	RCT, Italy	40, 36 (10), 100	BN (40) PNR group: 24.1 (SD = 6) TNR group: 24.0 (SD = 5.6)	20 (3)	20.6 (SD = 1.5)	Individual, 24 weeks	Psychobiological nutritional rehabilitation	20 (1)	21 (SD = 1.6)	Individual, 24 weeks

Abbreviations—AN: anorexia nervosa; BED: binge eating disorder; BN: bulimia nervosa; CBT: cognitive behavioural therapy; CNT: cognitive and nutritional therapy; COT: cognitive orientation treatment; DT: dietary therapy; EFT: emotion-focused therapy; NC: nutritional counselling; NR: not reported; PNR: psychobiological nutritional rehabilitation; RCT: randomised controlled trial; SD: standard deviation; SPT: self-psychological treatment; TNR: traditional nutritional rehabilitation.

**Table 2:** Components of intervention delivered by a dietitian in the dietetic arm of the study.

**Study**	**Specific Dietetic Intervention Components**	**General/Non-Dietetic-Specific Component**
Bachar 1999 [29]	Diet prescription tailored to fit patient preferences Education around meal regularity/scheduling and healthy eating Inclusion of foods that patients with BN would not usually binge on Advice to gradually acquire normal eating patterns to decrease binge/vomit episodes for patients with BN, and to increase meal frequency and calorie content for patients with AN	
Brambilla 2009 [30]	Nutritional advice without a specific diet	
Compare 2013 and 2016 [31,32]	Evaluation of nutritional status Nutrition therapy exploring obesity and its causes, correct nutritional choices, desirable body weight, preparing meals with different energy densities, calculating energy density using nutrition labels, using the energy-density formula and an energy-density value food chart Provision of sample meals, menus, and recipes	Strategies for practicing regular physical activity and for long-term weight management
Hall 1987 [33]	Restoration of normal eating patterns and dietary constituents Education around the relationship between eating behaviour and mood	Discussions about diet, mood, and daily behaviour patterns Guidance to increase patient confidence in maintaining control as weight gain occurred
Hsu 2001 [34]	Education covering good nutrition, nutritional requirements and the relationship between over-restrictive eating and binge eating Education on meal planning (including buying and preparing healthy food) to help establish and maintain regular eating patterns	
Laessle 1991 [35]	Instructions for keeping detailed nutritional diaries Analysis of nutritional diaries Structured eating that focused on appropriate meal timing (irrespective of appetite), adequate caloric intake, appropriate macronutrient composition and food variety. Advice to introduce fear/binge foods into daily eating Education about energy requirements, use of food-exchange lists for meal planning, and correcting misconceptions about specific foods Meal preparation and cooking advice Review of all strategies and relapse prevention	Psychoeducation about the relationship between restrained eating and binging Education about the physical consequences of binge eating and purging, the body’s ability to maintain a stable weight despite purging or improved eating behaviour, the psychological and biological effects of starvation, metabolic processes, and determinants of body weight and weight fluctuations Stimulus control techniques to avoid uncontrolled eating (e.g., not eating from a large packet) Encouragement to eat with others and a dinner with the therapists at a restaurant
Ruggiero 2003 [36]	Dietary management to help patients attain and maintain normal nutritional status (in adults) and normal growth (in adolescent), establish normal eating behaviour, promote normal attitudes towards food, and to assist patients in developing appropriate hunger and satiety signals Collection of dietary history Collaborative creation of nutritional plans consisting of regular, balanced eating with a minimum 1200 calories daily in the first week that gradually increased Use of dietary tools and substitutions to meet patient’s specific eating habits (e.g., substituting carbohydrates with vegetables, serving single-dish meals, providing semisolid foods)	Long-term psychoeducational treatment that aimed to achieve weight restoration
Serfaty 1999 [37]	Descriptions of normal eating patterns and basic food physiology Personalised modification to eating patterns	Psychological support provided by dietitian using supportive counselling
Sundgot-Borgen 2002 [38]	Education on principles of good nutrition, nutritional needs and the relationship between dieting and overeating Meal planning to establish and maintain regular eating	
Ventura 1999 [39]	Prescription of regular eating patterns (TNR only)	In both PNR and TNR background information about the multifactorial nature of EDs, sociocultural factors contributing to body image issues, medical complications associated with purging behaviours, set-point theory, consequences of dieting, relapse prevention and strategies to manage ED behaviours were provided PNR also (1) focused on how a network of interactions between psychobiological systems controls appetite and body weight and encouraged patients to try new ways of eating to “resynchronise an appetite system undermined… dieting”; (2) worked to help participants recognise hunger, appetite, and satiety cues; (3) encouraged participants to introduce a variety of macronutrients and notice the differing effects they had on satiety; (4) allowed participants to build their own meal plan.

Abbreviations—AN: anorexia nervosa; BN: bulimia nervosa; PNR: psychobiological nutritional therapy; TNR: traditional nutritional rehabilitation.

**Table 3:** Study outcomes.

**Study**	**Timepoints**	**ED Psychopathology**	**Other Psychopathology**	**Level of Function/** **Quality of Life**	**ED Behaviours/Weight**	**Diet** **Adequacy/** **Diet Quality**
Bachar 1999 [29]	Baseline, EoT	EAT 26: NSig within or between gps.	BSI: NSig within or between gps.	NR	NR	NR
Brambilla 2009 [30]	Baseline, EoT	EDI-2: Sig decrease within gp at EoT for Gp 1 but not Gp 2 or 3. Between gps NR.	SCL-90-R: Sig decrease within gp at EoT for Gp 1 total scores. Sig within gp at EoT for Gp 2 in subitems “depression” and “interpersonal relationships”. NSig within gp in Gp 3. Between gps NR.	NR	Binge frequency: Sig decrease within gp at EoT for Gp 1 patients but not Gp 2 or 3. Between gps NR.	NR
Compare 2013 and 2016 [31,32]	Baseline, EoT, 6-month FUp	BES: Sig decrease within gp at EoT and FUp in CT and EFT gps but not in DC gp. Between gps NR.	BUT: Sig decrease within gp at EoT and FUp for CT and EFT gps but not in DC gp. Between gps NR.	ORWELL-97: Sig decrease within gp at EoT and FUp in all gps. Between gps NR.	BES < 16: Sig within gp at EoT and FUp for CT, EFT but not DC. Between gps NR. Binge frequency: Sig decrease within gp at EoT and FUp for CT and EFT. NR in DC. Between gps NR. Weight: Sig decrease within gp at EoT and FUp for all gps. Between gps NR.	NR
Hall 1987 [33]	CCEI at baseline, EoT, 1-year FUp. Weight taken at baseline, 4× during treatment, 1-year FUp	CCEI (eating pattern score): Within gp sig NR. At FUp, PG > DAG in reducing symptoms of food avoidance and anxiety about eating with other people (sig NR). At FUp, DAG > PG in improving bulimia, vomiting, and purgation (sig NR).	CCEI (mental state score): Sig decrease within gp at FUp for dietary advice gp in somatic, phobic, and depression scales. Sig decrease within gp at FUp for psychotherapy gp in obsessional and depression scores. No between-gp differences.	CCEI (social adjustment score): Between-gp difference in social and sexual adjustment scores: PG > DAG.	Weight: Sig increase within gp at FUp for DAG only. No between-gp differences.	NR
Hsu 2001 [34]	Full assessment using all instruments assessed at baseline, week 6 of treatment, week 10 of treatment, and EoT Self-report measures and HDRS: week 6 of treatment and week 10 of treatment	EDI: Within gps sig NR. CNT > SG in reducing EDI subscales “drive for thinness”, “bulimia”, “ineffectiveness”, “perfectionism”, “interpersonal distrust”, and “interoceptive awareness”. CT > SG in EDI subscales “drive for thinness” and “ineffectiveness”. CNT > NT in reducing “drive for thinness”, “bulimia” and “ineffectiveness”. CT > NT only on the “bulimia” subscale. No between-gp differences between NT and SG, or between CT and CNT.	DAS: Sig within-gps decrease in DAS at EoT for all gps. Sig between-gp differences CT, CNT > SG. CNT > NT in decreasing DAS scores. No between-gp differences for CT and CNT.	SCS: Sig within-gp increases in SCS at EoT for all gps. Sig between-gp differences for self-control: CT, CNT, NT > SG. Sig between-gp differences in SCS: CNT > NT.	Binge frequency and vomit frequency: Sig within-gp decreases in binge and vomit frequency for all gps. No between-gp differences.	Meal pattern: Sig within-gp increase in number of meals eaten per day for all gps. No between-gp differences.
Laessle 1991 [35]	Baseline, week 3 of treatment, EoT, 6-month FUp, 12-month FUp	EDI: Sig decrease within gp at FUp for both gps. No between-gp differences.	BDI: Sig decrease within gp at FUp for BDI depression scores in both gps. Between groups NR.	NR	Binge frequency: Sig decrease within gp at EoT and FUp for both gps. No between-gp differences. Vomiting frequency: Sig decrease within gp at EoT for both gps. No between-gp differences.	Caloric intake: Sig increase in average amount of calories consumed in a day (outside of binges and not vomited) within gp at EoT and FUp in both treatments. No between-gp differences.
Ruggiero 2003 [36]	Baseline, 3 months into treatment, 6 months into treatment, 12 months into treatment	EDI: Sig within-gp decrease in “fear of fatness” in nutritional gp.	NR	NR	BMI: Sig within-gp increases in both gps. Pharmacological gp > nutritional treatment-only gp.	NR
Serfaty 1999 [37]	Baseline, 6 months into treatment, 6-month FUp	EDI: Sig within-gp decrease in CBT gp. Between groups N/A (100% DT attrition).	BDI: Sig within-gp decrease in CBT gp. Between groups N/A (100% DT attrition).	LCB: Sig within-gp decrease in CBT gp. Between groups N/A (100% DT attrition).	BMI: Sig within-gp increase in BMI in CBT gp. Between groups N/A (100% DT attrition).	NR
Sundgot-Borgen 2002 [38]	Baseline, EoT, 6-month FUp, 18-month FUp	EDI: No between-gp differences in “drive for thinness” or “body dissatisfaction” subscales at 18-month FUp. For “bulimia” subscale, CBT > NC at FUp.	NR	NR	Binge frequency: Within gps NR. Sig between-gp differences in reducing binge eating at FUp. Exercise gp > CBT Vomiting frequency: Within gps NR. Sig between-gp differences in reducing vomiting frequency, CBT > NC. Sig within-gp decrease in exercise gp.	NR
Ventura 1999 [39]	Monthly during treatment, 3-month FUp, 6-month FUp	NR	NR	NR	Binge frequency: Sig within-gp reduction in binge frequency for both gps. Between-gps PNR > TNR. Vomiting frequency: Sig within-gp reduction in vomiting frequency for both gps. Between-gps PNR > TNR.	Intake of carbohydrate servings: No between-gp differences. Intake of lipid servings (measured by serves of olive oil added): between gps PNR > TNR.

Abbreviations—BDI: Beck depression inventory; BES: Binge Eating Scale; BMI: body mass index; BUT: Body Uneasiness Test; CBT: cognitive behavioural therapy; CCEI: Crow-Crisp experiential index; CNT: cognitive and nutritional therapy; CT: combined therapy; DAG: dietary advice group; DAS: dysfunctional attitudes scale; DT: dietary therapy; EDI: Eating Disorders Inventory; EDI-2: Eating Disorder Inventory-2; EFT: emotion-focused therapy; EoT: end of treatment; FUp: follow-up; Gp: group; LCB: locus of control of behaviour; N/A: not available; NC: nutritional counselling; NR: not reported; NSig: no significance; NT: nutritional therapy; ORWELL-97: Obesity-Related Well-Being; PG: psychotherapy group; PNR: psychobiological nutritional rehabilitation; SCL-90-R: Symptoms Checklist-90-Revised; SCS: Self-Control Ccale Sig: significant; SG: support group; TNR: traditional nutritional rehabilitation.

**Table 4:** Summary of quality assessment conducted using MMAT (2018). Adapted from [28], with permission from authors, 2018.

	**Bachar [29]**	**Brambilla [30]**	**Compare [31,32]**	**Hall [33]**	**Hsu [34]**	**Laessle [35]**	**Ruggiero [36]**	**Serfaty [37]**	**Sundgot-Borgen [38]**	**Ventura [39]**
Screening Questions										
S1. Are there clear research questions?	Y	Y	Y	Y	Y	Y	Y	Y	Y	Y
S2. Do the collected data allow to address the research questions?	Y	Y	Y	Y	Y	Y	Y	N	N	Y
Quantitative randomised controlled trials										
2.1. Is randomisation appropriately performed?	CT	CT	NR	CT	CT	CT	NR	Y	CT	CT
2.2. Are the groups comparable at baseline?	Y	Y	NR	Y	Y	Y	NR	N	Y	Y
2.3. Are there complete outcome data?	Y	Y	NR	Y	Y	Y	NR	N	Y	Y
2.4. Are (participants, researchers and) outcome assessors blinded to the intervention provided?	CT	Y	NR	CT	N	N	NR	CT	CT	CT
2.5. Did the participants adhere to the assigned intervention?	CT	CT	NR	CT	Y	CT	NR	N	CT	Y
Quantitative non-randomised										
3.1. Are the participants representative of the target population?	NR	NR	CT	NR	NR	NR	Y	NR	NR	NR
3.2. Are measurements appropriate regarding both the outcome and intervention (or exposure)?	NR	NR	Y	NR	NR	NR	Y	NR	NR	NR
3.3. Are there complete outcome data?	NR	NR	Y	NR	NR	NR	CT	NR	NR	NR
3.4. Are the confounders accounted for in the design and analysis?	NR	NR	Y	NR	NR	NR	N	NR	NR	NR
3.5. During the study period, is the intervention administered (or exposure occurred) as intended?	NR	NR	CT	NR	NR	NR	CT	NR	NR	NR

Abbreviations—CT: cannot tell (unclear); N: no; Y: yes; NR = not relevant.

## Data Availability

Not applicable.

## Supplementary Materials

The following are available online at https://www.mdpi.com/article/10.3390/nu13124490/s1, Table S1: Search strategy used on the Ovid MEDLINE(R) ALL platform.

## Appendix A

**Table A1:** Search terms used in the systematic search of the electronic databases.

**Words Relating to Eating Disorders**	**Search Term Used**
Eating disorder Disordered eating Anorexia nervosa Anorexic Bulimia nervosa Binge eating disorder Binge eating Feeding disorder Orthorexia nervosa Muscle dysmorphia Rumination disorder * Purging disorder * Night eating syndrome Avoidant/restrictive food intake disorder (ARFID) Eating disorder not otherwise specified (EDNOS) Other specified feeding or eating disorder (OSFED) Unspecified eating or feeding disorder (UFED) Pica	Eating disorder * Disordered eating Anorexi * Bulimi * Binge Feeding disorder * Orthorexia Muscle dysmorphia Rumination disorder * Purging disorder * Night eating syndrome Intake disorder * ARFID EDNOS OSFED UFED Pica
**Words Relating to Dietitians**	**Search Term Used**
Dietitian Dietician Dietetic Nutritionist	Dieti#ian Dietetic * Nutrition *
**Words Related to Role in Treatment**	**Search Term Used**
Treatment Counselling Counsel Therapy Therapies Intervention Care Consultation Recommendation Plan Advice Management Education Prescription Support Role Function *	Treatment * Counsel * Therap * Intervention * Care Consult * Recommend * Plan Advice Management Educat * Prescri * Support Role * Function *

* Denotes a wildcard of any group of characters; # Denotes a wildcard of a single character.

## Appendix B

**Table A2:** PICO criteria.

**Population**
People with an eating disorder as defined by the Diagnostic and Statistical Manual of Mental Health Disorders 5th edition (DSM-5) [57]; any type (including but not restricted to anorexia nervosa, bulimia nervosa, binge eating disorder, avoidant/restrictive food intake disorder (ARFID), and other specified feeding or eating disorder (OSFED)). Any age, any gender, any ethnicity, and any severity of ED.
**Intervention**
Specialist dietetic care (meal support, nutrition counselling, nutrition education etc.) as part of treatment for an ED.
**Comparator**
Any other ED treatment modality.
**Outcome**
For each paper, outcomes measured by any validated instrument or questionnaire were selected for each of the following categories: Change in any eating disorder symptomology (e.g., Eating Disorder Inventory [59]; Eating Attitudes Test [60]); Change in other psychopathological measure (e.g., Beck Depression Inventory [61]; Depression Anxiety Stress Scales [62]); Change in functional outcome or quality of life (e.g., Quality of Life Scale [63]; Personal Wellbeing Index—Adult [64]); Change in nutritional status (e.g., BMI for individuals with AN; binge or purge frequency for individuals with BN); Change in diet quality (e.g., Australian Eating Survey [55]; Dietary Questionnaire for Epidemiological Studies [56]). If multiple outcome tools were used, reviewers selected the instrument that was either most consistently reported across studies or most researched. An exception to this was for the nutritional status category, where multiple outcomes were recorded if clinically significant (e.g., populations studied included both AN and BN, or binge and vomiting frequency were reported for BN).
**Study Design**
Controlled trials, randomised or non-randomised.
