# Supplementary material for: Beyond Refeeding: The Effect of Including a Dietitian in Eating Disorder Treatment. A Systematic Review
Source: Nutrients. 2021 Dec 15;13(12):4490. doi: 10.3390/nu13124490 (PMC8706437; doi:10.3390/nu13124490)
Supplement: Supplementary file 1 [file nutrients-13-04490-s001.zip › nutrients-1493617-supplementary.pdf]

Table S1: Search strategy used on the Ovid MEDLINE(R) ALL platform.

1	exp "Feeding and Eating Disorders"/
2	(eating disorder* or disordered eating or anorexi* or bulimi* or binge or feeding disorder* or orthorexia or muscle dysmorphia or rumination disorder* or purging disorder* or night eating syndrome or intake disorder* or ARFID or EDNOS or OSFED or UFED or PICA).ti. or (eating disorder* or disordered eating or anorexi* or bulimi* or binge or feeding disorder* or orthorexia or muscle dysmorphia or rumination disorder* or purging disorder* or night eating syndrome or intake disorder* or ARFID or EDNOS or OSFED or UFED or PICA).ab.
3	1 or 2
4	Dietetics/ or Nutritionists/
5	(dieti#ian* or dietetic* or nutrition*).ti. or (dieti#ian* or dietetic* or nutrition*).ab.
6	4 or 5
7	nutrition therapy/ or diet therapy/ or nutritional support/ or professional role/ or "scope of practice"/
8	(treatment* or counsel* or therap* or intervention* or care or consult* or recommend* or plan or advice or management or educat* or prescri* or support or role* or function*).ti. or (treatment* or counsel* or therap* or intervention* or care or consult* or recommend* or plan or advice or management or educat* or prescri* or support or role* or function*).ab.
9	7 or 8
10	3 and 6 and 9
